# Impact of oral and oropharyngeal cancer diagnosis on smoking cessation patients and cohabiting smokers

**DOI:** 10.18332/tid/109413

**Published:** 2019-11-01

**Authors:** Jenny L. Gray, Adnan Al Maghlouth, Hussain Al Hussain, Mohammed Al Sheef

**Affiliations:** 1 King Fahad Medical City, Riyadh, Saudi Arabia

**Keywords:** oral cancer, tobacco smoking, smoking cessation, oropharyngeal cancer, smoking-cessation advice

## Abstract

**INTRODUCTION:**

Our aim was to determine the influence of oral and oropharyngeal (OOP) cancer diagnosis on smoking cessation in patients and/or cohabiting smokers. We also aimed to determine whether OOP cancer patients received smoking-cessation advice and evaluated the factors that were influential in aiding them to quit or decrease smoking.

**METHODS:**

This study was conducted at King Fahad Medical City, Saudi Arabia from March 2015 to May 2017. A pre-validated self-administered questionnaire was administered to OOP cancer patients visiting the Dentistry and Head & Neck Oncology outpatient clinics. Sociodemographics and baseline information were obtained from electronic medical records. Data were collected from 203 patients; 88 were ever-smokers and 115 were never-smokers.

**RESULTS:**

Among patients who were smoking at the time of the OOP cancer diagnosis, 47.7% continued to smoke after the diagnosis. OOP cancer diagnosis was influential in smoking cessation in ever-smoker patients and their cohabiting smokers. The apparent influence of OOP cancer diagnosis was different between cohabiting smokers of ever-smoker patients (n=21/25; 84%) and those of never-smokers (n=10/21; 47.6%). Former-smokers (n=16/19; 84.2%) were less likely to remember receiving smoking-cessation advice than current-smokers (n=17/39; 43.6%). Pressure from family and friends, adverse impact on cancer prognosis, and adverse impact of cancer treatment were influential factors for smoking cessation. Among treatment modalities, combined chemoradiotherapy had the greatest impact (n=10/21; 47.6%) on smoking cessation among patients who stated that oncology treatment was influential in causing them to quit or decrease smoking.

**CONCLUSIONS:**

A substantial number of patients continued to smoke after the OOP cancer diagnosis; however, diagnosis facilitated smoking cessation in many cases. Current smoking status should be reviewed throughout the OOP cancer patient’s disease course, and smoking-cessation assistance should be provided where necessary.

## INTRODUCTION

Approximately 20% of all cancer deaths are attributed to smoking^1^. Tobacco smoking has been shown to cause oral cancer^2-4^. Approximately 75% of lip, oral cavity, and pharyngeal cancers are attributable to tobacco smoking and alcohol^5^. Oral cancer risk decreases by about 35% within 1–4 years of smoking cessation, with reduction of about 80% by 20 years of cessation. Even after oral cancer diagnosis, smoking cessation is important to improve survival rates; patients who continue to smoke are at higher risk of cancer recurrence and show poorer response to treatment than those who quit before treatment (relative risk=2.5, 95% CI: 1.4–4.4)^6^. The risk of someone having oral cavity or pharyngeal cancer during their lifetime is approximately 1.2%^7^, and there is a considerable difference between the estimated lifetime risk for men and women (1.85% vs 0.37%). This gender disparity is the same for most countries because of the differences in exposure to risk factors (i.e. tobacco, alcohol, sunlight, etc.)^6^. However, over the past few decades, the gender disparity for those diagnosed with OOP cancer has declined (at present approximately 1.5:1 for oral and 2.8:1 for oropharyngeal cancers)^6^.

Globally, there are approximately 300400 new cases of oral cancer and 145400 deaths from oral cancer per year^8^. From a recent report on smoking, it is estimated that 23.7% male and 1.5% female Saudi adults use tobacco, which is approximately 16 times as many male as female smokers^4^. Most countries are currently experiencing a downward trend in the prevalence of smoking^9,10^ and global age-standardized prevalence estimates have drastically decreased by approximately 25% between 1980 and 2012. However, Saudi Arabia is experiencing the opposite with approximately a 2% ‘annualized rate’ increase, which makes it the highest rate of change among 187 countries and one of only six countries with a significant increase in the prevalence of smoking^11^.

Tobacco contains up to 50 different types of known carcinogens that are in direct contact with the oral cavity during the smoking process^12^. WHO estimates that the prevalence in smoking among Saudi males may reach 36% by 2025^13^. Saudi Arabia has the additional risk of OOP cancer from commonly used cultural carcinogens, such as qaat (a green shrub), sheesha (waterpipe) and shamma (smokeless tobacco), particularly in certain regions of Saudi Arabia such as Najran and Jaizan, which are large areas in the south of Saudi Arabia. Most previous studies concerning smoking in the Saudi population have been conducted on specific, easily accessible populations, such as students and healthcare workers. A systematic review of smoking in Saudi Arabia, found that a considerable percentage of smokers acknowledged that they wanted to quit but were unable to do so^14^.

It is estimated that approximately 2% of middle-aged smokers are able to stop annually with no help, which is far below the approximate two-thirds of smokers who express a wish to stop, half of whom try to stop within any given year. Depending on the level of professional support and intervention, approximately 2–19% of smokers will be able to successfully quit smoking^15^.

There is limited information addressing the smoking status of patients with OOP cancer in Saudi Arabia and there is no study on the Saudi population addressing the impact of an OOP cancer diagnosis on the patient’s smoking status, although this topic has been addressed previously in various other populations^16-28^. In a retrospective analysis^29^ of secondhand smoke, it was estimated that approximately 34% of adult non-smokers were exposed to secondhand smoke. In the Eastern Mediterranean region (including Saudi Arabia) this figure is closer to 24.5%. The study showed that female non-smokers were at least 50% more likely to die from the effects of secondhand smoke than their male counterparts, as they are more likely to be non-smokers than men (about 60% more female non-smokers than men)^29^. This figure may be a very modest estimate for Saudi Arabia, considering the huge gender disparity among smokers^29^.

Therefore, the present study aimed to determine to what extent an OOP cancer diagnosis influences a smoker, whether it influences other cohabiting smokers, the number of OOP cancer patients who recall being given smoking-cessation advice previously, and whether a specific cancer treatment modality influenced smokers to quit or decrease smoking.

## METHODS

### Study design and setting

This was a cross-sectional study. The information was gathered from a self-administered questionnaire survey and other sociodemographic data were taken from the electronic medical records. The questionnaire was administered to patients at King Fahad Medical City, a large, tertiary-care hospital in Riyadh, Saudi Arabia, between March 2015 and May 2017. For the purpose of this study, OOP cancer includes that of the lips, tongue, floor of the mouth, palate, parotid gland, oropharynx, nasopharynx and larynx.

### Subjects

Ever-smokers were classified as those who had previously smoked cigarettes, conversely never-smokers were those who reported never to have smoked a cigarette previously (the lowest estimated number of cigarettes smoked by an ever-smoker in our sample was four cigarettes per day for two years). A questionnaire in Arabic was administered to every patient, visiting the Dentistry clinics, with a documented pathological diagnosis of oral or oropharyngeal cancer. For those few patients who we were unable to reach through the Dentistry clinics we were able to collect the questionnaire from them at the neighboring Head & Neck Oncology outpatient clinics of the same institution. The minimum acceptable age of a patient at the time of answering the questionnaire was set at 16 years, with no maximum age limit. Nineteen patients were excluded. The exclusion criteria included those patients who were younger than 16 (n=14), those who did not speak Arabic (n=3), and those patients who refused to take part (n=2). The study duration was from March 2015 to May 2017. The study was approved by the Institutional Review Board at King Fahad Medical City (IRB 15-083). Each patient was asked for verbal consent to participate in the study.

### Study instrument

A self-administered questionnaire was formulated. The questionnaire included questions about their current health status, if they had ever previously smoked, if there were other smokers cohabiting with them and the impact, if any, that the OOP cancer diagnosis had on them or their smoking cohabiter. The second section of the questionnaire was specifically for ever-smokers, asking about their current smoking status, when they last smoked, if they had received smoking-cessation advice previously and what has been the biggest influence on them in relation to decreasing smoking or cessation. The questionnaire was translated into Arabic by experts and back-translated from Arabic to English. A panel of experts in dentistry, oncology, and research, determined the psychometric characteristics (test/re-test reliability) of the back-translated version with the original, which was then piloted on the first 20 patients. The questionnaire was completed by the patients during the recording of vital signs by nurses, who had been trained to give a brief explanation of the questionnaire’s purpose. The questionnaires were collected, and the data were uploaded daily into an excel sheet. Sociodemographics and baseline information were obtained from the electronic medical records. No questionnaires returned were deemed invalid but several were incomplete related to sociodemographic information from the electronic medical record such as occupation in 29 patients and education level in 57 patients.

### Measures and variables

The following variables were evaluated: age, gender, education, employment status, OOP type, OOP site, smoking-related, cumulative amount of smoking (pack-years). Questions were asked regarding current smoking status of patients and smoking-cessation advice received. Regarding cohabiting smokers, questions regarding whether diagnosis affected them and in what way they were affected. To investigate whether there was an association between pack-years, education, and employment type (controlling for age), a linear regression analysis was performed with pack-year as the outcome and age, education (recoded as college or more vs less or unknown), and employment (recoded in three categories: 1. no, housewife, student; 2. unknown; and 3. employed) as the factors. Gender could not be used as a factor owing to the lack of information on pack-years among the few female smokers in the sample population. We also investigated if there was a relationship between the site of the cancer (e.g. nasopharynx, oral cavity, and larynx) and smoking cessation (excluding patients who quit smoking >6 months before the diagnosis, assuming that they quit smoking before any obvious symptoms). To estimate the effect on cohabiting smokers, we asked: 1) if the diagnosis had affected them, and 2) in what way (e.g. quit, decreased, and tried to quit).

### Statistical analysis

Categorical variables (e.g. gender, smoking status) are presented as frequencies and percentages, whereas continuous variables (e.g. age) are expressed as mean ± standard deviation (SD). Chi-squared/Fisher’s exact tests were applied based on whether the expected cell frequency was <5, applied to determine the significant association between categorical variables. Two-tailed p-values of <0.05 were considered as statistically significant. Correlation between pack-years with age, education and employment status were evaluated, apart from those between cancer site and smoking cessation, cohabiting smokers’ smoking history and recollection regarding smoking-cessation advice among ever-smokers and never-smokers. A logistic regression analysis was performed with smoking status and gender, age, education, cancer site and stopping smoking. All data were entered and analyzed using the statistical package SPSS 25 (SPSS Inc., Chicago, IL, USA).

## RESULTS

### Survey responses

In total, 203 questionnaires from 115 never-smoker patients and 88 ever-smoker patients were analyzed (Table 1). There were 222 patients with OOP cancer who attended the outpatient clinics during the duration of the study, the questionnaire was returned by 203 patients, two declined to answer and 17 did not meet the inclusion criteria. The mean age at diagnosis was 50.2 years for ever-smokers and 44.7 years for never-smokers. The gender difference was evident with ever-smokers, as 93 (94.3%) were male and only 5 (5.7%) were female, while among never-smokers the number of male respondents were 49 (42.6%) and female were 66 (57.4%).

**Table 1:** Sociodemographic characteristics of the sample (n=115)

*Parameter*	*Eversmokers*	*Neversmokers*
	*n (%)*	*n (%)*
**Gender**		
Male	83 (94.3)	49 (42.6)
Female	5 (5.7)	66 (57.4)
**Nationality**		
Saudi	75 (85.2)	108 (93.9)
Non-Saudi	13 (14.8)	7 (6.1)
Minimum–maximum age at diagnosis	14–86	16–84
Mean age at diagnosis (SD)	50.2 (14.1)	44.7 (15.6)
**Cancer site**		
NPC	41 (46.6)	54 (46.9)
Oral cavity	22 (25)	42 (36.5)
Laryngopharynx	19 (21.6)	8 (7)
Salivary gland	2 (2.3)	8 (7)
Other	4 (4.5)	3 (2.6)
**Employment status**		
Employed	46 (52.3)	38 (33)
Retired	19 (21.6)	8 (7)
Unknown	18 (20.4)	20 (17.4)
Unemployed	2 (2.3)	2 (1.7)
Student	2 (2.3)	8 (7)
Housewife	1 (1.1)	39 (33.9)
**Educational status**		
Illiterate	8 (13.8)	8 (10.4)
Elementary (up to grade 6)	9 (15.5)	11 (14.3)
Intermediate (grades 7–9)	9 (15.5)	3 (3.9)
High school (grades 10–12)	10 (17.2)	24 (31.2)
University	22 (37.9)	31 (40.3)

Logistic regression analysis of the correlation of patient sociodemographic factors with smoking status as the outcome is provided in Table 2. The odds of a male patient being an ever-smoker was almost twenty-fold that of the female counterparts (OR=19.9, 95% CI: 7.7–62.3, p<0.001), which is similar to the discrepancy between Saudi male and female smokers. Smokers were likely to be older males (OR=1.02, 95% CI: 1.00–1.05, p=0.04). None of the predictors for current smoking status (gender, age, employment status or education) was significantly correlated with smoking cessation. After controlling for age, higher education was marginally not associated with a lower number of pack-years for male smokers (education less than college (correlation coefficient= -17.5, 95% CI: -36.0 to -1.0, p=0.06); female smokers were excluded because only one female ever-smoker had pack-years information). After excluding patients who had given up smoking >6 months prior to the diagnosis, the association between cancer site and stopping smoking was significant (p=0.002), with patients with laryngeal cancer being much more likely to stop.

**Table 2:** Logistic regression analysis with dependent variables the smoking status and the number of pack-years and independent variables the demographic characteristics

*Parameter^1^*	*OR*	*95% CI*	*P*
Gender (Ref: female)	19.9	7.7–62.3	<0.001
Age	1.02	1.00–1.05	0.04
Education (Ref: less than college)	0.77	0.33–1.75	0.52
Employment status: (Ref: no)			
Unknown	1.26	0.46–3.46	0.66
Employed	1.34	0.59–3.03	0.48
***Parameter^2^***	***OR***	***95% CI***	***P***
Gender (Ref: female)	1.16	0.14–7.75	0.88
Age	1.02	0.98–1.05	0.35
Education (Ref: less than college)	0.71	0.22–2.33	0.56
Employment status: (Ref: no)			
Unknown	0.60	0.15–2.30	0.45
Employed	1.55	0.52–4.58	0.43

OR: odds ratio, CI: confidence interval.

1 Current smoking status

2 Number of pack-years

We initially performed a logistic regression analysis of current smoking status, sex, age, education, and employment status; the analysis did not reveal any significant predictors. Therefore, among smokers, the odds of quitting do not change according to sex (95% CI: 0.14–7.75, p=0.88), age (95% CI: 0.98–1.05, p=0.35), education (95% CI: 0.22–2.33, p=0.56), or employment status (95% CI: 0.52–4.58, p=0.43) (Table 3). In the linear regression analysis for the association of pack-years with other factors, after controlling for age, a higher education was marginally associated with a lower number of pack-years. Analysis of the relationship between the cancer site and smoking cessation, after excluding the patients who quit <6 months before their OOP cancer diagnosis, showed that the association between cancer site and smoking cessation was significant (p=0.002).

**Table 3:** Logistic regression analysis with dependent variable the pack-years and independent variables age, education and employment status

*Parameter*	*Correlation coefficient*	*95% CI*	*P*
Age	0.61	(0.09, 1.13)	0.02
Education (Ref: less than college)	−17.5	(−36.0, −1.0)	0.06
Employment status (Ref: no)			
Unknown	−10.7	(−32.5, 11.1)	0.33
Employed	−5.0	(−22.2, 12.2)	0.55

OR: odds ratio, CI: confidence interval.

### Patients’ smoking status trends, relatives’ smoking status, effect of oral cancer diagnosis

Of the ever-smokers, 4 (4.5%) had an unknown current smoking status, while among the remaining, 21 (25%) continued smoking and 63 (75%) had quit. From those who had quit smoking, 20 patients (31.8%) had quit smoking >6 months before the diagnosis (Table 4), with smoking duration ranging from 1–50 years (mean 18.3 years) before the diagnosis.

**Table 4:** Smoking status trends regarding cancer diagnosis and treatment

*Ever Smokers*	*n*	*%*
**Smoking status**		
Quit > 6 months prior to diagnosis	28	31.8
Quit < 6 months before diagnosis	12	13.6
Quit after diagnosis	11	12.5
Quit during treatment	11	12.5
Quit after treatment finished	1	1.1
Still smoking	21	23.9
Unknown	4	4.5

Among the ever-smokers, 4 (4.5%) did not respond whether there was another smoker living with them, 25 (28.4%, p=0.087) indicated that they were cohabiting with another smoker, and the remaining 59 (67%) reported that there was no other smoker living with them (Table 5). Among the 25 patients with cohabiting smokers, one patient (4%, p=0.450) was unsure whether the cancer diagnosis had affected the other smoker, 21 (84%, p=0.009) acknowledged that the cancer diagnosis had affected the other smoker(s) in some way, and 3 (12%, p=0.018) reported that it had not. Approximately 8 (38%, p=0.314) smoking-cohabiters were able to quit; in addition, 4 (19%, p=0.213) were trying to quit, another 4 (19%, p=0.139) were trying to decrease smoking, and 2 (9.5%, p=0.045) managed to decrease smoking (Table 2).

**Table 5:** Comparison between cohabits of ever-smoker families and cohabits of never-smoker families regarding the effect of cancer diagnosis on them

	*Ever smoker family n (%)*	*Never smoker family n (%)*	*P*
**Do you cohabit with another smoker?**			
Yes	25	21	0.087
**Was there any effect on cohabiting smokers?**			
Yes	21 (84)	10 (47.6)	0.009
No	3 (12)	9 (42.8)	0.018
Unknown	1 (4)	2 (9.5)	0.450
**If yes, what was the effect?**			
They quit	8 (38.1)	2 (20)	0.314
They tried to quit	4 (19)	4 (40)	0.213
They tried to reduce the amount	4 (19)	0	0.139
smoked			
They successfully reduced the	2 (9.5)	4 (40)	0.045
amount smoked			
Unknown	3 (14.3)		0.209

Among the never-smokers, 10 patients (8.7%) did not respond whether there was a smoker cohabiting with them, 21 (18.2%) indicated that there was another smoker cohabiting with them, and the remaining 84 (73%) indicated that there was no other smoker cohabiting with them. Among the 21 never-smoker patients with cohabiting smokers, the diagnosis had an impact on 10 (47.6%), two patients (9.5%) cited an unknown impact and the remaining 9 (42.8%) said that it did not have an impact. Moreover, in the never-smoker patient’s household 40% cohabiting smokers had tried to quit, 20% had successfully quit, and the remaining 40% had decreased the amount of smoking owing to their family member’s cancer diagnosis (Table 5).

Approximately 67% of cohabiting smokers were apparently affected by the cancer diagnosis of their family member. However, the results varied after stratification of the data according to the never-smokers and ever-smokers. Although there were almost twice as many smoking-cohabitants of smokers than those of never-smokers, 84% family members of ever-smokers were affected in some way, whereas only 47.6% family members of never-smokers claimed to be affected (Table 2). Among the ever-smoker group, 25% (n=21) continued to smoke even after the OOP cancer diagnosis and its subsequent treatment; this value increased to 46.5% when patients who had given up smoking >6 months before the diagnosis were excluded. From those who continued smoking, 55% said that they now smoked less, 10% said that there was no change in the amount they smoked, and the remaining did not answer this question. Furthermore, among the current-smokers, 70% responded that they had received smoking-cessation advice, 5% responded they had not received any, 5% responded that they could not remember, and the remaining 20% did not answer the question (Table 6). Furthermore, when asked what had been the biggest influence on them with regards to smoking cessation, 37.5% responded that they tried to quit due to pressure from family and/or friends, 25% responded that they thought that smoking would make the cancer worse, 18.75% responded that they were concerned that smoking would negatively impact on the outcome of the cancer treatment, and the remaining 18.75% responded that they thought the cancer was caused by their smoking. Twenty-one (24.1%) patients stated that cancer treatment(s) had made them quit or decrease smoking, with the most common reason being chemoradiotherapy.

**Table 6:** Descriptive statistics for the recall of smoking-cessation advice

*Do you remember receiving professional smoking-cessation advice?*	*Current smoker*		*Former smoker*		*p*
	*n*	*%*	*n*	*%*	
Yes	16	84.2	17	43.6	0.003
No	2	10.5	10	25.6	0.182
Unsure	1	5.3	12	30.8	0.029

### Smoking-cessation advice trends

Among ever-smokers, 58 patients answered the question regarding whether they had received smoking-cessation advice previously. Thirty-four patients (58.6%) responded that they had received smoking-cessation advice previously, 12 (20.7%) were unsure if they had, and 12 patients (20.7%) responded that they had not received any smoking-cessation advice. Current-smokers were more likely than former-smokers (84.2% vs 43.6%, p=0.003) to recall being given smoking-cessation advice (Table 4).

## DISCUSSION

The primary aim of this study was to investigate how many ever-smoker patients diagnosed with OOP cancer would continue smoking after cancer diagnosis. The current smoking status of patients with OOP cancer must be frequently ascertained by the healthcare providers who come in contact with these patients and appropriate assistance given if they are found to be still smoking. In all, 47.7% of ever-smokers who were smoking in the 6 months prior to the OOP cancer diagnosis continued to smoke after the diagnosis and the treatment, which is more than double the average prevalence of Saudi smokers. Approximately 22% of patients quit smoking after the diagnosis but before starting cancer treatment, indicating that the diagnosis was enough for them to immediately quit smoking. A similar percentage of patients were able to quit during cancer treatment, with chemoradiotherapy being cited as the main treatment modality that caused this change. It has been previously hypothesized that those quitting smoking within a few months before the diagnosis do so because of alterations in the OOP condition, which make smoking uncomfortable^20^. In our survey, 13.6% of the patients fit into this category.

It has been noted that countries such as Saudi Arabia, that have a high Human Development Index (HDI; an amalgamated statistic based on life expectancy, education level and per capita income), have a lower incidence of OOP cancer, as shown by the latest GLOBOCAN statistics, where the age-standardized rate is 3.9 per 100000 males compared to 8.7 in low/medium HDI countries^30^. A previous systematic review of tobacco use and head–neck oncology also showed that a substantial number of patients with oral cancer continue to smoke after their diagnosis, with a mean prevalence of 57.3%, which is slightly higher than the findings from our relatively small study^31^. Globally, the incidence of lip, oral cavity and pharyngeal cancers account for just <4% of all cancers but this is predicted to rise by an astonishing 62% by the year 2035^5^.

Although oral cavity cancer incidence rates have been declining globally, probably due to the concurrent decrease in smoking rates, certain areas, for example South Asia, due to factors such as predicted population growth and use of oral carcinogens like betel and bidi cigarettes, the burden of lip, oral cavity, and pharyngeal cancers is predicted to rise. In some Asian countries, oral cancer is the most common cancer in men, accounting for ≤35% of all cancer incidences. Oral cancer risk increases with age, whereas we found the mean age of diagnosis to be 44.7 years for never-smokers and 50.2 years for ever-smokers, other studies seem to show that globally the average age of diagnosis is substantially higher, for example between 2000 and 2004 in the US the median age was 62 years, with only 6% oral cancers occurring in those under 45 years^6^.

The relative risk of recurrent oral cancer is higher among current-smokers compared to previous-smokers (2.9), this goes up to 3.8 for those current-smokers who smoke >2 packs per day. In one study of head and neck cancer patients, approximately half were still smoking after one year of treatment, 30% of those went on to have a second primary compared to only 13% of those patients that had quit smoking^32^. The risk of squamous cell carcinoma of the head and neck for cigarette smokers is approximately 10-fold the risk for never-smokers^3^.

As observed in our study, although smokers are aware of the risks related to smoking, they frequently continue smoking^33^. A previous study conducted in Saudi Arabia showed a high level (73–95%) of awareness of the dangers associated with smoking^7^. Although no local data are currently available regarding the relationship between smoking cessation and an OOP cancer diagnosis, studies conducted in other countries show that 35–54% of smokers will continue to smoke even after an OOP cancer diagnosis^10,34^. Another study reported that increased cessation rates were more likely to occur in patients with higher education, those diagnosed at later stages, those with laryngeal cancer in particular, and those having surgery as part of their cancer treatment^23^. The cessation rates were 53–96%, with most studies showing that approximately one-third of patients with head and neck cancer continue to smoke after the diagnosis^31^. In our study, logistic regression analysis and linear regression analysis showed that higher education was marginally associated with a lower number of pack-years, and patients with laryngopharyngeal cancer were more likely to quit. The number of ever-smokers who continue smoking is relatively high probably because of two main factors: first, the patient may not acknowledge that smoking has any correlation to the cancer; second, smoking is a well-known addiction and although the patient may assume that smoking has had a causative role, they may be unable to quit smoking during this particularly stressful time.

We analyzed the influences on smoking cessation in cancer patients. Pressure from family and friends, adverse impact on cancer prognosis, and adverse impact of cancer treatment were reported as influences on quitting smoking. However, although many patients provided different factors that influenced them to try to quit smoking, unfortunately the reasons that they cited were not sufficient to enable them to quit smoking. The effects of cancer treatment (radiation and chemotherapy) had an influence on smoking cessation in approximately one-quarter of the patients. Although 21 patients stated that cancer treatment had made them quit or decrease their smoking, no inference could be drawn from this as to whether either treatment modality (chemotherapy or radiotherapy) encouraged patients to quit or decrease smoking. This is because many head and neck cancers were treated with concurrent chemotherapy and radiotherapy and because of the relatively small sample size. Various cancer treatments, particularly radiation therapy and surgery, seem to have an impact on smokers toward decreasing or quitting smoking^35^, although it is not known how much of this is attributable to the treatment modality and how much is attributable to an OOP cancer diagnosis.

In our study, among ever-smokers, cancer diagnosis had a smoking-cessation or smoking-reduction effect in the patients as well as on cohabiting smokers. A significantly higher proportion (87.5%) of family members of ever-smokers were affected by cancer diagnosis, compared to only 52.6% family members of never-smokers. This may be an indication of the lower perception of the role of secondhand smoke as a causative factor among cohabiting smokers. Among the cohabiters of ever-smokers, 45% were able to quit smoking whereas only 20% cohabiters of never-smokers were able to quit. A similar, but weaker, trend was seen among the never-smokers.

In our study, current-smokers were more likely than former-smokers (84.2% vs 43.6%) to recall being given smoking-cessation advice. This is probably because of two main reasons: the length of time between the questionnaire and the patient giving up smoking (they may have forgotten whether they were advised) and current-smokers were more likely to be asked by the healthcare professionals about their current smoking status. In our opinion, the discrepancy between the numbers of ever-smokers that recall receiving smoking-cessation advice may be explained by the relatively long mean number of years that the patient had given up smoking (18.3 years); they may not recall advice given that many years previously.

Health professionals are in an ideal position to provide smoking-cessation advice to all patients, particularly to those at risk of or with certain medical conditions that are exacerbated by smoking. However, several studies show that because of various reasons, many healthcare professionals do not use this opportunity to provide smoking-cessation advice. In one study, only 33.2% of Saudi healthcare practitioners felt that they were adequately trained to provide smoking-cessation advice^24^. One review estimated that only 20% of oncologists provide health-promotion advice^36^. With approximately 64% of patients with cancer now surviving >5 years, which accounts for 3–4% of the total US population, a greater effort is needed to encourage them to live a healthier life, particularly considering that patients who have had cancer die of non-cancer diseases at a higher rate than the general population, for example, almost half of cancer survivors die from cardiovascular disease^8^.

### Limitations

This study has several limitations. First, we faced difficulty in finding the smoking status of patients at various stages of disease or treatment, smoking may be regarded as more of a fluid concept, with possible relapses as it is very addictive; some patients with OOP cancer may quit smoking within the duration of their cancer treatment and re-start smoking once they view themselves to be cancer-free. Second, we did not attempt to assess alcohol consumption with our patients, combined with smoking, alcohol intake is estimated to have an 80% attributable risk for oral cancer; people who are both heavy drinkers and heavy smokers have 38 times the oral cancer risk than total abstainers. As alcohol consumption is illegal in Saudi Arabia, we were concerned that patients may not be truthful in answering any questions to do with alcohol consumption^6^. Moreover, objective markers for current smoking status were not employed, and patients smoking history (where available) was calculated as pack-years. However, it has been suggested that not only is the overall number of cigarettes smoked important but also the duration, i.e. that fewer cigarettes over a longer time frame is a higher risk factor for oral cancer than more cigarettes over a shorter period^37^. Furthermore, due to the lack of testing equipment, there was no biochemical validation of smoking cessation. It has been noted that self-reporting generally leads to some underestimation of smoking prevalence, by approximately 6%^25^, particularly for patients with diseases that could be feasibly linked to their smoking status^26^. Finally, we did not evaluate the HPV status of patients, as it is not routinely tested for in our institution. Therefore, the data regarding HPV was a very small sample. Considering that HPV is implicated in the etiology of head and neck cancer, this was a limitation in our study.

## CONCLUSIONS

Even with a diagnosis of OOP cancer, a high number of current smoker patients will continue to smoke even after being diagnosed. Therefore, healthcare professionals cannot be complacent with OOP cancer patients, by focusing solely on treating the disease or assessing for signs of recurrence. There needs to be more of a proactive approach toward continually reevaluating the ever-smoker patients’ current smoking status and supporting the patient in any smoking-cessation efforts^38^. It has been shown that even short interactions with patients, advising them about smoking cessation can have an effect^15^. Smoking cessation will improve treatment outcomes and lessen the risk of recurrence.
